# Ionic and Enzymatic Multiple-Crosslinked Nanogels for Drug Delivery

**DOI:** 10.3390/polym13203565

**Published:** 2021-10-15

**Authors:** Qian Tao, Julong Zhong, Rui Wang, Yuzhu Huang

**Affiliations:** School of Chemistry and Materials Science, Ludong University, Yantai 264025, China; zhongjulong@163.com (J.Z.); wangrcherry@163.com (R.W.); hyuzhu741@sina.com (Y.H.)

**Keywords:** multiple-crosslinked nanogel, ionic crosslink, enzymatic crosslink, drug delivery, natural polymeric materials

## Abstract

Both ionic and enzymatic crosslink are efficient strategies for constructing network materials of high biocompatibility. Here chitosan was modified firstly and then crosslinked by these two methods for complementary advantages. The preparation methods and ionic crosslinkers can regulate the size and uniformity of the multiple-crosslinked nanogels. The multiple-crosslinked nanogels with the smallest size and the best uniformity was selected for the drug delivery. The drug-loading content and encapsulation efficiency were up to 35.01 and 66.82%, respectively. Their release behaviours are correlated with the pH value and the drug dosage. In general, the lower pH value and the lower drug dosage promoted the drug release. With the assistance of several kinetic models, it is found that drug diffusion plays a preponderant role in drug release, while polymer relaxation has a subtle effect. The multiple-crosslink resulting from ionic compounds and enzymes may provide a new perspective on developing novel biocompatible materials.

## 1. Introduction

Natural polymeric materials are popular candidates for drug carriers due to their excellent biocompatibility and biodegradability. Many strategies have been devised to endow the drug carriers based on natural polymeric materials with extensive and advanced functions [1,2,3]. The stable and compact structures composed of natural polymers are generally desired. Various kinds of crosslinkers play vital roles in building these structures [4,5]. The chemical crosslinking reagents (e.g., dialdehyde) were introduced early and performed satisfactorily. However, the possible cytotoxicity [6] and strong covalent bonding brought by chemical crosslinkers impair the biocompatibility and biodegradability of natural polymeric materials. More attention has been paid to bio-friendly crosslinkers, such as ionic crosslinkers and enzymatic crosslinkers.

Ionic crosslinking is a mild reaction that is suitable for natural polymers [7,8,9]. Chitosan becomes a polycationic polymer in a solution of a pH value less than 6. Thus it can be crosslinked by the anionic reagent, such as divalent sulphate (SO_4_^2−^), trivalent phosphate (PO_4_^3−^), and pentavalent tripolyphosphate (TPP). Ionic crosslinked materials often exhibit excellent biocompatibility and pH sensitivity, making them qualified for drug carriers [10]. The enzyme can catalyze the crosslinking of natural polymers efficiently. The amino acid residues on the gelatin chains are crosslinked rapidly with the catalysis of microbial transglutaminase (mTG) [11,12,13]. The phenol groups grafted on the natural polymers can be crosslinked in the presence of horseradish peroxidase (HRP) and H_2_O_2_ [14,15,16]. The enzymatic reactions occur fast and gently under the physiological environment. More significantly, the biocompatibility of enzymes is beyond doubt [17,18].

However, the ionic crosslinked materials are hard to retain stable structures, and enzymatic crosslinked materials may degrade quickly. It is realized that the combined utilization may complement crosslinking methods for each other and obtain a material with optimized properties. The combinations of crosslinking methods have been reported in the studies of tissue engineering scaffolds [19,20]. For example, the interpenetrating polymer network (IPN) materials are composed of two different polymers crosslinked by two different methods [21]. The IPN hydrogels prepared by Lee et al. were composed of chitosan and poly (acrylic acid) [22]. The IPN hydrogels reported by Li et al. were composed of gelatin and alginate [23].

Nevertheless, one polymer multiple-crosslinked by several methods is more favourable as drug carriers with nanoscales because of the easier preparation and more controllable delicate structures. Here we crosslinked the same polymer by ionic and enzymatic crosslinkers coordinately to prepare nanogels whose structures were easily regulated. Their performances in a drug delivery were studied furthermore. The novel multiple crosslinking is expected to bring a new perspective for the drug carriers with natural polymer.

## 2. Materials and Methods

### 2.1. Materials and Instruments

Chitosan (the deacetylation degree ≥ 95%), 1-(3-Dimethylaminopropyl)-3-ethylcarbodiimide hydrochloride (EDC-HCl), N-hydroxysuccinimide (NHS), Phloretic acid (PA), 2-(*N*-Morpholino)ethanesulfonic acid (MES), Sodium tripolyphosphate (TPP), and Sodium molybdate (Na_2_MoO_4_) and horseradish peroxidase (HRP) (>300 U/mg, lyophilized powder) were all purchased from Aladdin Biochemical Technology Co., Ltd. (Shanghai, China) and used as received. 5-Fluorouracil (5-FU) was obtained from Macklin Biochemical Co., Ltd. (Shanghai, China). Nuclear magnetic resonance (NMR) spectrometer (AVANCE NEO, 500 MHz, Bruker) and Fourier transform infrared (FTIR, MAGNA550, Nicolet) was used for structural analysis. Nanoparticle size analyzer (Nano-ZS90, Malvern) was used to determine the size of the nanogels. UV-Vis spectrophotometer (T6, Persee) was used to determine the concentrations of 5-FU in the drug loading and release experiments.

### 2.2. Synthesis of Phenolic Hydroxyl Modified Chitosan (MC)

The phenolic hydroxyl group was conjugated to the amine group of chitosan in the presence of EDC/NHS. 1.28 g of chitosan was dissolved in 100 mL of a 0.5% acetic acid solution [24,25,26]. The pH value was adjusted to approximately 5.0 after complete dissolution. PA of 0.88 g, 0.93 g of NHS, and 1.52 g of EDC-HCl were dissolved in a 100 mL mixture of N, N-dimethylformamide (DMF) and deionized water (*v/v* = 3:2). Then it was blended with a chitosan-acetic acid solution and kept at ambient temperature for 20 h. The reaction mixture was dialyzed against deionized water for several days and then freeze-dried to obtain the modified chitosan (MC). ^1^H NMR spectrum of MC in D_2_O was recorded at 500 MHz. FTIR spectra of MC and chitosan were recorded in the wavenumber range of 4000–400 cm^−1^ using KBr pressed disk technique.

### 2.3. Preparation of Nanogels with TPP and HRP Multiple-Crosslinking

The MC was dissolved in MES solutions for at least 24 h, and the pH values were kept at 5.0. TPP and HRP were respectively dissolved at specific concentrations in advance. Then the chitosan nanogels were prepared according to the composition listed in Table 1. Besides the different TPP concentrations, different preparation methods were also employed. One method was adding the MC solution into the TPP solution first and then mixing it with the HRP and H_2_O_2_ solution. The other method was adding the MC solution into the TPP, HRP, and H_2_O_2_ mixed solution. All the solution mixing and the preparations of the nanogels were performed with mild electromagnetic stirring and stabilized at room temperature for 24 h.

### 2.4. Preparation of Nanogels with Na_2_MoO_4_ and HRP Multiple Crosslinking

The nanogels with Na_2_MoO_4_ and HRP were prepared according to the method above, and their composition was listed in Table 2.

### 2.5. Measurement of the Nanogel Size

The nanogel size was determined by a nanoparticle size analyzer with the appropriate dilution. Measurements of all the samples were repeated 3 times, and the hydrodynamic diameter distribution was recorded based on the scattered light intensity.

### 2.6. Drug Loading

5-FU was dissolved in MES solutions and subsequently mixed with the MC solution. The mass ratio of 5-FU to MC was 50, 100 and 150%, respectively. These mixtures were used to prepare drug-loaded nanogels, referring to the method above. The solutions of nanogels were dialyzed against deionized water for 3 h. Then the concentrations of 5-FU in dialysates were determined by UV-Vis spectrophotometer. The standard solutions of 5-FU were prepared in the concentrations of 2.5 mg/L, 5 mg/L, 7.5 mg/L, 10 mg/L, 12.5 mg/L, respectively. The UV-Vis spectra of 5-FU solutions showed the highest absorption peak at 265 nm, which was selected as the measuring wavelength to determine the standard curve of 5-FU. The standard calibration was repeated 3 times, and the R^2^ of the standard curve were all more than 0.99. The drug-loading content (DLC%) and entrapment efficiency (EE%) of the nanogels were calculated according to the reference [27].

### 2.7. Drug Release

The drug release in vitro was studied by UV-Vis spectrophotometer. The drug-loaded nanogels (5 mL) were dialyzed against 50 mL of MES solutions at 25 °C. The pH value of the MES solution was adjusted in advance to 5.5, 6.5 or 7.5 by adding hydrochloric acid or potassium hydroxide. A 3 mL portion of the dialysate was extracted at predetermined intervals to determine the concentration of the drug. At the same time, 3 mL of fresh MES solution was added to the dialysate. The experiments were performed in triplicate, and the results were expressed in terms of the cumulative drug release [28].

The kinetics of the 5-FU released from the nanogels was determined by fitting the release profiles to several theoretical models (including zero-order, first-order, Higuchi, Korsmeyer-Peppas, and Peppas-Sahlin models).

## 3. Results and Discussion

### 3.1. Structural Characterization and Enzymatic Crosslinking of MC

The ^1^H NMR spectrum of MC (Supplementary Materials Figure S1) showed the chemical shifts of both PA groups and chitosan, proving that the phenolic hydroxyl was grafted on the chitosan. The chemical shifts at 6.76 and 6.44 ppm corresponded to the aromatic protons of the phenolic group. The chemical shifts at 2.44 and 2.27 ppm corresponded to the methylene protons of PA. The chemical shifts from 4.51 to 2.80 ppm corresponded to the glucopyranose ring protons of chitosan. FTIR was also used to confirm the chemical structure (Figure S2). In the FTIR spectrum of chitosan, the bands at 3354 cm^−1^ (stretching vibrations of –OH and –NH_2_ in chitosan), at 1150 cm^−1^ (asymmetric stretching vibration of C–O–C bridge between glucopyranose rings), at 1024 cm^−1^ (stretching vibrations of glucopyranose ring) were observed. The FTIR spectrum of MC exhibited the bands attributing to the stretching vibration of –OH in the phenolic group (3257 cm^−1^), asymmetric stretching vibration of C–H in the methylene of PA (2920 cm^−1^), and stretching vibration of C–C in the phenolic group (1644–1514 cm^−1^).

**Figure 1:**
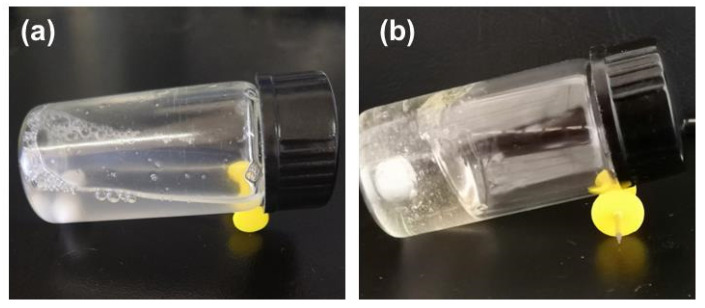
The MC solution before (**a**) and after (**b**) HRP-catalysed crosslinking. The concentrations of MC, HRP, and H_2_O_2_ are 10 mg mL^−1^, 5 U mL^−1^, and 0.8 mM.

The crosslinking of the MC under the enzymatic catalysis was verified. The MC solution was low-viscous and flowable before adding HRP, whereas the gel was formed soon after adding HRP (Figure 1). It proves that the phenolic hydroxyl groups were grafted with the chitosan chains, which linked to each other in the presence of HRP. Thus, a three-dimensional network was constructed. This enzymatic crosslinking was in line with expectations and ready for the preparation of multiple-crosslinked nanogels.

### 3.2. Influences on the Preparations of Multiple-Crosslinked Nanogels

**Figure 2:**
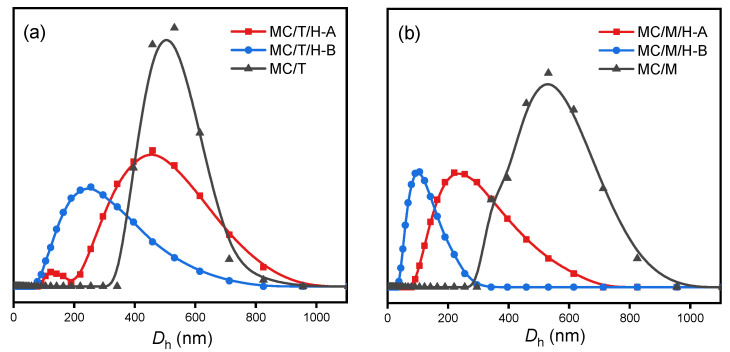
Hydrodynamic diameter distributions of multiple-crosslinked nanogels prepared with different methods. The concentrations of TPP (**a**) or Na_2_MoO_4_ (**b**) were 0.05 mg mL^−1^, respectively.

Two methods were used to prepare the multiple-crosslinked nanogels, where the crosslinking occurred with the ionic crosslinker first and then with HRP (method A) or the crosslinking occurs with the ionic crosslinker and HRP simultaneously (method B). Besides, the nanogels prepared only with ionic crosslinkers were compared with them (Figure 2).

The different hydrodynamic diameters of the nanogels indicate that the HRP-involved nanogel generally has a smaller size because the extra crosslinking provided by HRP makes the chitosan networks more compact. Further comparing methods A and B shows that the nanogels prepared by method B were smaller than those prepared by method A. it is considered that the crosslinking of chitosan chains is more efficient when two types of crosslinkers coexist. It is tricky for HRP to catalyze in method A because the ionic crosslinking occurs first, making a part of the phenolic hydroxyl groups wrapped inside.

In addition to the method, the type of ionic crosslinker also significantly impacts the preparation of multiple-crosslinked nanogels. The multiple-crosslinked nanogels prepared with Na_2_MoO_4_ have a smaller size and narrower distribution, which is more competitive for drug delivery. The crosslinking efficiency depends on the ionic bonds between anions of the crosslinker and amino ions of the chitosan. TPP exists as a pentavalent anion, and Na_2_MoO_4_ exists as a divalent anion in solutions. TPP possesses more joinpoints than Na_2_MoO_4_ with the same mass. Hence more chitosan chains were linked with TPP rather than Na_2_MoO_4_, causing the larger nanogels.

**Figure 3:**
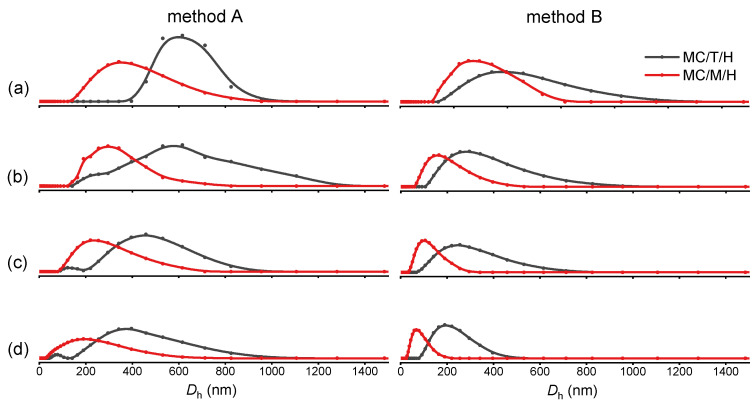
Hydrodynamic diameter distributions of multiple-crosslinked nanogels prepared with the method A and B. The concentrations of TPP or Na_2_MoO_4_ were 0.20 (**a**), 0.10 (**b**), 0.05 (**c**), 0.04 (**d**) mg mL^−1^, respectively.

Moreover, the concentrations of ionic crosslinkers have an apparent effect on the size of the multiple-crosslinked nanogels. The sizes of the nanogels decrease as the ion concentration decreases, shown in both methods A and B (Figure 3). The higher ion concentration provides more crosslinking points and thus increases the possibility of forming larger crosslinking structures. It is noted that the nanogels prepared by method B were all smaller than those prepared by method A in the case of four different concentrations.

Considering the above influences, the multiple-crosslinked nanogel labelled as MC/M/H-B4 was selected for the subsequent drug delivery experiments due to its smallest size and the best uniformity among all the samples.

### 3.3. Drug Loading in Multiple-Crosslinked Nanogels

In the drug delivery experiments, 5-FU was selected as a model drug. 5-FU is a common anti-metabolism and anti-tumour drug, but it usually causes gastrointestinal discomfort when taken directly. Therefore, a sustained-release carrier is necessary to improve drug utilization and reduce the damage to healthy cells.

**Figure 4:**
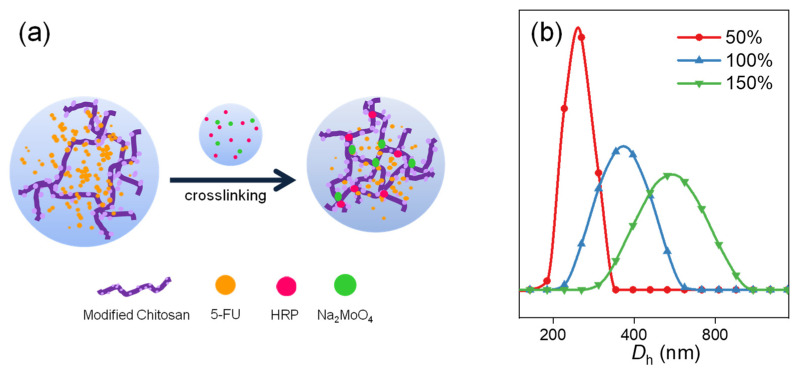
(**a**) Scheme of 5-FU loading in multiple-crosslinked nanogel; (**b**) Hydrodynamic diameter distributions of MC/M/H-B4 nanogels with different 5-FU dosages.

The drug-loading method determined by screening tests is to mix 5-FU firstly with MC rather than with crosslinkers. This method is feasible because the 5-FU could be dispersed evenly among the complex spatial conformation of chitosan longer chains, improving the loading efficiency of 5-FU in the network structures of nanogels (Figure 4a).

The size and uniformity of the drug-loaded nanogels are necessary for the long-term circulation of drug carriers in the body. Here the influence of different drug dosages was studied (Figure 4b). With the increase of drug dosage from 50 to 150%, the hydrodynamic diameters of 5-FU loaded nanogels increase, and the size distributions become wide.

The essential criteria for drug-loading experiments are drug-loading content (DLC) and entrapment efficiency (EE), listed in Table 3. The DLC increased from 23.55 to 35.01% with the drug dosage increase. However, the increase gets small when the dosage increases from 100 to 150%. It is indicated that increasing the drug dosage would not be worthwhile because the DLC almost reached the maximum amount that the nanogels could withstand. The EE decreased with the drug dosage increase and reached 66.82% when the drug dosage was 50%. Overall, increasing the drug dosage could achieve a higher DLC, but more 5-FU cannot be loaded in the carrier, resulting in a waste of drugs. The balance between the DLC and EE should be determined on the requirements of specific applications.

### 3.4. Drug Release from Multiple-Crosslinked Nanogels

**Figure 5:**
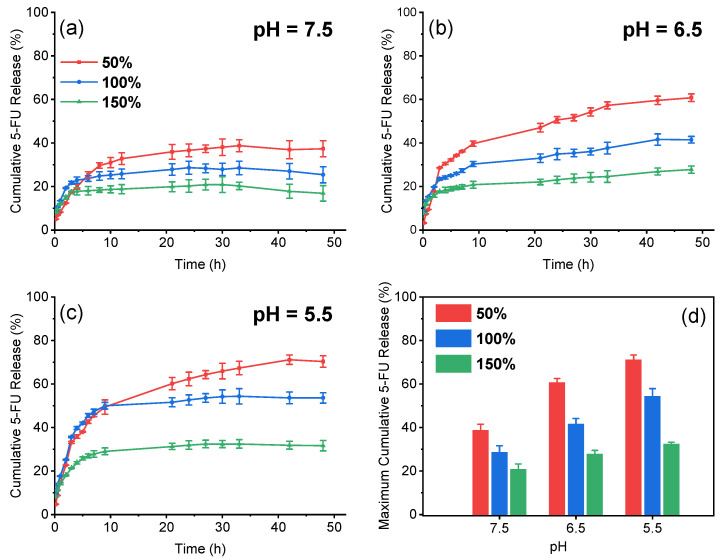
Release profiles of MC/M/H-B4 nanogels (**a**–**c**) and the maximum cumulative 5-FU release from nanogels with different drug dosages at different pH values (**d**).

Chitosan shows pH sensitivity due to the amino groups. Consequently, the release profiles of chitosan-derived drug carriers are generally impacted by the pH values. The influences of pH on drug release behaviours from MC/M/H-B4 nanogels were studied (Figure 5).

The 5-FU release was slow in the first 30 h at pH 7.5, and the maximum cumulative release was 38.7% (dosage of 50%). The 5-FU duration increased to 48 h at pH 6.5, and the maximum cumulative release was 60.8% (dosage of 50%). The fastest 5-FU release occurred at pH 5.5, and the maximum cumulative release was 71.2% (dosage of 50%). It shows that the drug could be released from the MC/M/H-B4 nanogels and be easier to leave at lower pH values. As the pH value decreases, the drug release becomes faster, and the maximum cumulative release increases.

The reasons for the pH-responsive release were analyzed. The protonated chitosan chains are more stretched and bring looser networks, causing faster drug release at a lower pH. However, 5-FU was also protonated in the acid medium and competed with chitosan. It is difficult for chitosan chains to maintain the loose structure when the 5-FU concentration increases. Therefore the two higher dosages groups’ release at pH 5.5 slowed down significantly after 20 h.

It is also found that the drug release becomes quicker, and the maximum cumulative release increases with the drug dosage decrease at the three different pH levels. (Figure 5d). The path of drug diffusion is complicated due to the network structure. Thus the drug molecules are more difficult to arrive outside when the drug-loading content is higher.

Furthermore, several kinetic models were used to evaluate the drug release profiles for revealing the mechanism of drug release. The equations and parameters of the different models are listed in Table 4 [29,30,31]. The *R*^2^ of Korsmeyer-Peppas and Peppas-Sahlin are higher than zero-order, first-order and Higuchi, indicating that Korsmeyer-Peppas and Peppas-Sahlin are adequate for describing the release behaviours.

The diffusional release exponent (*n*) of the Korsmeyer-Peppas model reveals the mechanism of drug release from porous hydrophilic polymers and is effective when the released drug is no more than 60%. Moreover, the *K*_2_ of the Peppas-Sahlin model represents the polymer relaxation’s contribution to the drug release with no restriction on the drug release [32]. Therefore, a combination of these two models is optimal to analyze the chitosan-derived nanogels.

The *n* values for the 100 and 150% dosage are less than 0.45, indicating that these release mechanisms are Fickian diffusion. The *n* values for a dosage of 50% are more than 0.45 and less than 0.89, indicating a case II transport (erosion-controlled drug release) [33]. The *n* values increase with pH decrease. It is considered that the hydrogen bonds between the chitosan and 5-FU are formed. The proportion of 5-FU that forms hydrogen bonds is more prominent with less dosage, and the lower pH is conducive to hydrogen bonding.

*K*_1_ and *K*_2_ of Peppas-Sahlin models respectively represent drug diffusion and polymer relaxation contributions to the drug release [34]. For all release profiles of 5-FU release from MC/M/H-B4 nanogels, drug diffusion plays a preponderant role in the drug release rate, while polymer relaxation has a subtle effect on the release rate.

## 4. Conclusions

The ionic (TPP or Na_2_MoO_4_) and enzymatic (HRP) crosslinkers were employed to prepare the multiple-crosslinked chitosan nanogels. The size and uniformity of the multiple-crosslinked nanogels are influenced by preparation methods and types and concentrations of the ionic crosslinker. The multiple-crosslinked nanogels of small size and high uniformity were selected for the drug delivery. The multiple-crosslinked chitosan nanogels perform impressively on drug loading and controlled release. The drug-loading content and encapsulation efficiency are up to 35.01 and 66.82%, respectively. Adjusting the pH value and the drug dosage could change the release rate and the maximum cumulative release from the multiple-crosslinked nanogels. Moreover, the evaluation of kinetic models explains the release mechanism under different conditions.

The ionic and enzymatic multiple-crosslinked nanogels positively impact biocompatibility and work well as drug carriers. The method developed in this study could provide new opportunities for biomedical applications of natural polymers.

## Figures and Tables

**Table 1:** Composition of multiple-crosslinked nanogels with TPP and HRP.

Sample	MC (mg mL^−1^)	TPP (mg mL^−1^)	HRP ((U mL^−1^)	H_2_O_2_ (mM)
MC/T/H-A1	1.0	0.20	0.5	0.08
MC/T/H-A2	1.0	0.10	0.5	0.08
MC/T/H-A3	1.0	0.05	0.5	0.08
MC/T/H-A4	1.0	0.04	0.5	0.08
MC/T/H-B1	1.0	0.20	0.5	0.08
MC/T/H-B2	1.0	0.10	0.5	0.08
MC/T/H-B3	1.0	0.05	0.5	0.08
MC/T/H-B4	1.0	0.04	0.5	0.08

A: The crosslinking occurs with TPP first and then with HRP; B: The crosslinking occurs with TPP and HRP simultaneously.

**Table 2:** Composition of multiple-crosslinked nanogels with Na_2_MoO_4_ and HRP.

Sample	MC (mg mL^−1^)	Na_2_MoO_4_ (mg mL^−1^)	HRP (U mL^−1^)	H_2_O_2_ (mM)
MC/M/H-A1	1.0	0.20	0.5	0.08
MC/M/H-A2	1.0	0.10	0.5	0.08
MC/M/H-A3	1.0	0.05	0.5	0.08
MC/M/H-A4	1.0	0.04	0.5	0.08
MC/M/H-B1	1.0	0.20	0.5	0.08
MC/M/H-B2	1.0	0.10	0.5	0.08
MC/M/H-B3	1.0	0.05	0.5	0.08
MC/M/H-B4	1.0	0.04	0.5	0.08

A: The crosslinking occurs with Na_2_MoO_4_ first and then with HRP; B: The crosslinking occurs with Na_2_MoO_4_ and HRP simultaneously.

**Table 3:** The drug-loading content and entrapment efficiency of MC/M/H-B4 nanogels for 5-FU (all data are expressed as mean ± SD, *n* = 10).

	*m*_0_*/m* (%)		
	50	100	150
**DLC (%)**	23.55 ± 4.68	32.30 ± 7.04	35.01 ± 5.19
**EE (%)**	66.82 ± 7.92	64.60 ± 4.09	58.35 ± 8.66

*m*_0_ and *m* are the weight of 5-FU and MC/M/H-B4 nanogels, respectively.

**Table 4:** Kinetic parameters of 5-FU release from MC/M/H-B4 nanogels.

Models	*m*_0_*/m* (%)	pH = 5.5			pH = 6.5			pH = 7.5		
		*R* ^2^			*R* ^2^			*R* ^2^		
Zero-order	50	0.9108			0.8786			0.9365		
*F* = *kt*	100	0.8967			0.8655			0.7143		
	150	0.8902			0.7734			0.647		
		*R* ^2^			*R* ^2^			*R* ^2^		
First-order	50	0.9496			0.9081			0.9538		
*F* = 1 − *e^−kt^*	100	0.9314			0.8881			0.7322		
	150	0.9077			0.7902			0.6562		
		*R* ^2^			*R* ^2^			*R* ^2^		
Higuchi	50	0.9856			0.9647			0.9874		
*F* = *kt*^0.5^	100	0.9768			0.9661			0.8765		
	150	0.982			0.9149			0.8276		
		*R* ^2^	*n*		*R* ^2^	*n*		*R* ^2^	*n*	
Korsmeyer-Peppas	50	0.9908	0.6106		0.9798	0.6502		0.9767	0.4823	
*F* = *kt^n^*	100	0.9849	0.4493		0.9893	0.3026		0.9536	0.2738	
	150	0.9947	0.3302		0.9687	0.2369		0.9444	0.2199	
		*R* ^2^	*k*_1_	*k*_2_	*R* ^2^	*k*_1_	*k*_2_	*R* ^2^	*k*_1_	*k*_2_
Peppas-Sahlin	50	0.9947	0.3034	−0.0286	0.9853	0.2361	−0.0206	0.9851	0.1933	−0.0208
*F = k*_1_ *t^m^ + k*_2_ *t* ^2*m*^	100	0.9717	0.3067	−0.0385	0.9809	0.1161	−0.0094	0.9764	0.1333	−0.0174
	150	0.9901	0.1568	−0.0194	0.9542	0.0686	−0.0062	0.9565	0.0935	−0.0132

In all models, *F* is the fraction mass of 5-FU released at time *t*, and *k* (or *k*_1_, *k*_2_) is the kinetic constant.

## Data Availability

Data is contained within the article.

## Supplementary Materials

The following are available online at https://www.mdpi.com/article/10.3390/polym13203565/s1, Figure S1: ^1^H NMR spectrum of MC in D_2_O at 500 MHz, Figure S2. FTIR spectra of chitosan and MC.
