# Supplementary material for: Ionic and Enzymatic Multiple-Crosslinked Nanogels for Drug Delivery
Source: Polymers (Basel). 2021 Oct 15;13(20):3565. doi: 10.3390/polym13203565 (PMC8541401; doi:10.3390/polym13203565)
Supplement: Supplementary file 1 [file polymers-13-03565-s001.zip › polymers-1411154-supplementary.pdf]

polymers

Supplementary Material

Ionic and Enzymatic Multiple-crosslinked Nanogels for Drug Delivery

Qian Tao *, Julong Zhong, Rui Wang and Yuzhu Huang

School of Chemistry and Materials Science, Ludong University, Yantai 264025, China
* Correspondence: taoqian@ldu.edu.cn

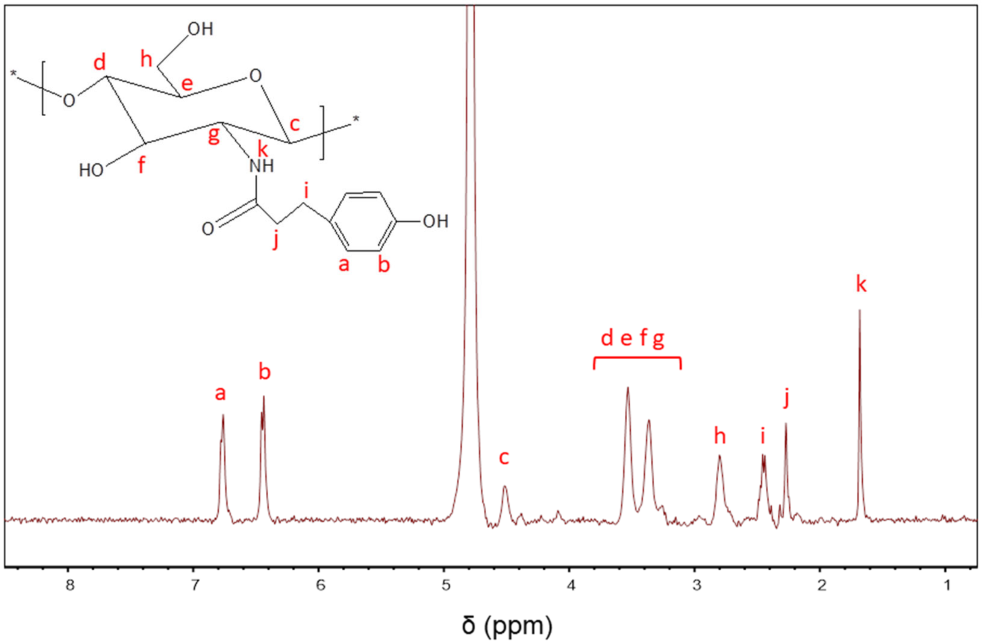

Figure S1. ^{1}H NMR spectrum of MC in D_2O at 500 MHz.

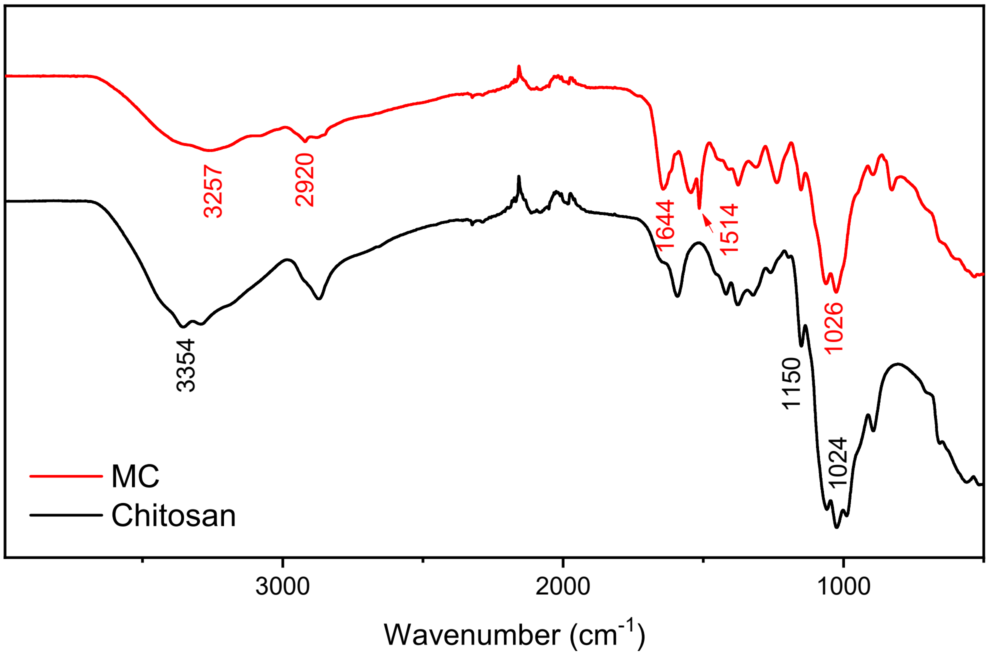

Figure S2. FTIR spectra of chitosan and MC.
